# Comparison of phenolic content and antioxidant activities of millet varieties grown in different locations in Sri Lanka

**DOI:** 10.1002/fsn3.415

**Published:** 2016-08-24

**Authors:** Disna Kumari, Terrence Madhujith, Anoma Chandrasekara

**Affiliations:** ^1^ Department of Applied NutritionWayamba University of Sri LankaMakandura, GonawilaSri Lanka; ^2^ Department of Food Science and TechnologyUniversity of PeradeniyaPeradeniyaSri Lanka

**Keywords:** Finger millet, foxtail, proso, Sri Lanka

## Abstract

Soluble and bound phenolic compounds were extracted from different varieties of millet types namely, finger millet, foxtail, and proso millet cultivated at dry and intermediate climatic zones in Sri Lanka. The extracts were examined for their total phenolic content (TPC), total flavonoid content (TFC), and proanthocyanidin content (PC). The antioxidant activities were meassured by reducing power (RP), trolox equivalent antioxidant capacity (TEAC), 2,2‐diphenyl‐1‐picrylhydrazyl (DPPH) radical scavenging activity, ferrous ion chelating ability (FICA), and using a β carotene linoleate model system. The ferulic acid content of extracts were determined using high‐performance liquid chromatoghraphy (HPLC). Finger millet showed the highest phenolic content and antioxidant activities compared to proso and foxtail millets. The phenolic content as well as antioxidant activites of soluble and bound phenolic extracts of millets were affected by variety and cultivated location. The highest phenolic content and antioxidant activites were reported for millet samples cultivated in areas belonging to the dry zone in Sri Lanka.

## 1 Introduction

Cereals play a vital role in human diet as an important source of energy, protein, and micronutrients among others for majority of people in the world. Dietary recommendations worldwide emphasize the significance of cereals in a balanced diet. Furthermore, cereals have been proven to provide additional health benefits while satisfying the energy and nutritional needs of humans. Risk of non‐communicable diseases (NCDs) is increasing worldwide at an alarming rate in developed as well as developing regions. Several studies found that the regular consumption of whole grains and wholegrain products are helpful to prevent and to reduce the prevalence of NCDs (Okarter & Liu, 2010; Slavin, 2004).

Cereals have been used as staple foods both directly for human consumption and indirectly via livestock feeding since the ancient times. Cereal grains commonly cultivated for foods include wheat, rice, maize, oats, rye, barley, sorghum, and millets; the latter include a wide array of small‐seeded grains. Millets are at the sixth place in world cereal production. They are the major food source for people living in economically disadvantaged status in Africa and Asia. Millets are known as the first domesticated cereals that were cultivated at the beginning of human civilization.

Different millet types include brown top (*Panicum ramosum*), Japanese barnyard (*Echinochloa crusgalli*), finger millet (*Eleusine coaracana*), proso millet (*Panicum miliaceum*), kodo millet (*Paspalum scrobiculatum*), little millet (*Panicum sumatrense*), pearl millet (*Pennisetum glaucum*), and foxtail (*Setaria italica*) millet. Pearl millet is the most widely cultivated grains globally among these millet species at present (Annor, 2013).

Millets are being recognized as potential future crops due to their nutrient contents similar to other major cereals and non ‐nutrient compounds having proven health benefits. Studies have shown that millet grains are rich sources of non ‐nutrients, especially phenolic compounds (Chandrasekara & Shahidi, 2010, 2011; Varsha, Asna, & Malleshi, 2009). There are evidences to show that phenolic compounds can act as antioxidants within the human body to protect against oxidative stress and to reduce the risk of NCDs (Chandrasekara & Shahidi, 2011; Shobana, Ushakumari, Malleshi, & Ali, 2007).

Plants produce phenolic compounds in response to stress conditions such as infections, wounding, and UV radiation, among others. Especially, the environmental factors such as sun exposure, soil type, and rainfall have an effect on phenolic content of plants (Manach, Augustin, Morand, Remesy, & Jimenez, 2004). In addition to environmental factors cultivated location, growing season and cultivar also have influenced the phenolic, and flavonoid contents of buckwheat seeds (Oomah & Mazza, 1996).

There are 46 agro‐ecological regions based on rainfall conditions, temperature, elevations, and soil conditions in Sri Lanka. Millets are cultivated at a number of locations in different agro‐ecological regions. Proso, foxtail, and finger millets are common among the other millet types growing in Sri Lanka. Finger millets are at the third place of cereal production and are cultivated over 7,000 hectares in Sri Lanka. Common three finger millet varieties, namely, *Ravi*,* Ravana*, and *Oshada* are used in Sri Lanka in addition to other local varieties. This study was aimed to determine the content of phenolic compounds and their antioxidant activities of millet types, namely finger, proso, and foxtail millets, grown in different locations in Sri Lanka.

## 2 Materials

### 2.1 millet samples

Seventeen millet grain samples grown in different locations in Sri Lanka were used in this study. Three types of millets, namely finger millet (*Elusine coracana*), proso millet (*Panicum miliacium*), and foxtail millet (*Setaria italica*) were included. Table 1 presents agro‐ecological regions and cultivated locations of the samples used in this study. Names of samples used in the study were given based on the cultivated location and the varietal name. Four varieties, namely *Ravi*,* Ravana*,* Oshada*, and local variety of finger millets were obtained from the Field Crops Research and Development Institute, Mahailluppallama, Sri Lanka, and Palwehera seed collecting center, Agriculture Department of Sri Lanka. Samples of foxtail millet and proso millet were obtained from Agriculture School, Palwehera and local farmers.

**Table 1:** Millet varieties and cultivated locations in Sri Lanka

Millet sample^a^	Variety	Agro‐ecological zone	Cultivated location
Finger millet			
MIRavi	Ravi	DL1	Mahaillupallama
MIRav	Ravana	DL1	Mahaillupallama
MIOsh	Oshada	DL1	Mahaillupallama
MOL	Local	IL2	Moneragala
AML	Local	DL2	Ampara
HAML	Local	DL5	Hambanthota
WAL	Local	IL3	Wariyapola
THAL	Local	DL1	Thabuththegama
ALUOsh	Oshada	IL2	Aluttharama
NIKOsh	Oshada	IL3	Nikaweratiya
BATRav	Ravana	DL5	Bataatha
Foxtail millet			
PALL	Local	DL1	Palwehera
HAML	Local	DL5	Hambanthota
MOL	Local	IL2	Moneragala
JAFL	Local	DL3	Jaffna
Proso millet			
ANUL	Local	DL1	Anuradhapura
HAML	Local	DL5	Hambanthota

(DL‐ Dry zone, Low country; IL – Intermediate zone, Low country).

a Given name of millet sample is based on cultivated location and variety.

### 2.2 Chemicals

Folin‐Ciocalteu's reagent, linoleic acid, tween 80 (polyoxyethylene sorbitan monopalmitate), and sodium nitrite were purchased from Research Lab Fine Chem Industries, Mumbai, India. Vanillin, 2,2‐diphenyl‐1‐picrylhydrazyl (DPPH), 2,2 azinobis (3‐ethylbenzothia zoline‐6‐sulfonate diammonium salt) (ABTS), 2,2‐azobis(2‐methylpropionamidine) dihydrochloride (AAPH), β‐carotene, ferrous chloride, sodium chloride, butylated hydroxyanisole (BHA), ferulic acid, trolox, catechin, diethyl ether, ethylacetate, methanol, and acetone were purchased from Sigma‐Aldrich, St Louis, USA. Sodium carbonate, ferric chloride, were purchased from Thomas Baker (Chemicals) Limited, Bombay, India.

Aluminum chloride, trichloroacetic acid (TCA), and potassium phosphate dibasic were purchased from Techno Pharm Chem, India. Sodium hydroxide, potassium phosphate monobasic, and potassium hydroxide, were purchased from Loba Chem Pvt Ltd, India. 3‐(2‐pyridyl)‐5, 6‐diphenyl‐1,2,4‐triazine‐4,4‐disulfonic acid sodium salt (Ferrozine) were purchased from SERVA Electrophoresis GmbH, Heildberg, Germany. Ethylene diamine tetra acetic acid tri sodium salt (Na_3_EDTA) was purchased from Needham Market Sufflock, England.

## 3 Methods

### 3.1 Determination of proximate composition of millet grains

Moisture, ash, crude protein, crude fiber, and crude fat contents of raw, dehulled millet grain samples were determined according to the AOAC (1999) methods.

### 3.2 Sample preparation

Whole grains of millets were dehulled to separate hulls from grains. Finger millet grains were dehulled using a rice polishing machine (Rice husker and polisher PM 500, Satake Engineering Co Ltd, Japan). Foxtail millets and proso millets were dehulled using rice milling machine (Rice machine, Satake Engineering Co Ltd, Japan). Whole grains, dehulled grains, and hulls were separately used for the extraction of phenolics. Samples were ground using a blender (Phillips HR 2011, Koninklijke Phillips Electronics N.V., China) and sieved using a siever with 0.038 seive opening (As 200, Retsch, Germany). Samples were defatted using hexane (1:5 w/v, 2 min two times) at ambient temperature. Defatted samples packed in polythene pouches were stored at −80°C until used within a week for extraction of phenolic compounds.

### 3.3 Extraction of soluble phenolic compounds

Soluble phenolic compounds were extracted from whole grains, dehulled grains, and hulls of millets. Defatted meal (5 g) was mixed with 100 ml of 70% (v/v) aqueous acetone and then placed in a water bath maintained at 60°C and stirred at maximum speed for 25 min under refluxing conditions with magnetic stirrer (KMC 130SH, Vision Scientific Co Ltd). The resultant slurry was centrifuged for 5 min at 3,000*g* (Refrigerated centrifuge 3‐18R TOMOS Life Science Group, USA) and supernatant was collected. The extraction procedure was repeated for two times. Combined supernatants were evaporated in rotary evaporator (IKA RV‐10, IKA^®^‐Werke GmbH & Co. KG, Germany) at 40°C at 125 rpm. Concentrated samples were freeze dried at −55°C, and 12 × 10^‐3^ mbar (Alpha 1‐4 LD plus CHRIST, Germany). Lyophilized crude phenolic extracts were stored at −80°C until used for further analysis. The residues of all samples were air‐dried for 12 hr and stored at −80°C until used for the extraction of bound phenolic compounds. During all stages, extracts were protected from light by covering with aluminum foil.

### 3.4 Extraction of bound phenolic compounds

Bound phenolic compounds were extracted as explained by Chandrasekara and Shahidi (2010). In brief, the residues obtained after extraction of soluble phenolic compounds were hydrolyzed using 2 mol/L NaOH for 4 hr, stirring at room temperature in a shaking water bath (BT 680D, YIH DER Co., Ltd, Taiwan) at ambient temperature under a nitrogen atmosphere. The pH of the resulting slurry was adjusted to pH 2 with 6 mol/L HCL. Diethyl ether and ethyl acetate (1:1, v/v) were used to extract the bound phenolic compounds. The extraction was done in three times and desolventized to dryness at 30°C in a rotary evaporator (IKA RV‐10, IKA^®^‐Werke GmbH & Co. KG, Germany). Known volumes of methanol were used to reconstitute the phenolic compounds and stored at −80°C for further analysis.

### 3.5 Determination of total phenolic content

The total phenolic content (TPC) of each extract was determined using Folin‐Ciocalteu's reagent (Chandrasekara & Shahidi, 2010). Folin‐Ciocalteu's reagent (0.25 ml) was added to 0.25 ml of methanolic extracts of soluble phenolics (2 mg / ml) in a centrifuge tube. The contents were vortexed and 0.5 ml of saturated sodium carbonate was added. After adding 4 ml of distilled water, the reaction mixture was kept in dark for 35 min, at room temperature and followed by centrifugation for 10 min at 4000*g*. The absorbance of the supernatant was measured at 725 nm (UV‐VIS Spectrophotometer, Labomed Inc, USA). Appropriate blanks were used for background subtractions. A standard curve prepared using ferulic acid was used to determine the TPC as μmol ferulic acid equivalents (FAE) / g of dry matter (dm).

### 3.6 Determination of total flavonoid content

The total flavonoid content (TFC) was determined by a colorimetric method (Chandrasekara & Shahidi, 2010). The extracts of soluble phenolic compounds were dissolved in methanol to obtain a concentration of 2 mg / ml. The centrifuge tube containing 0.5 ml of soluble phenolic extract was added 2 ml of distilled water and 0.15 ml of 5% NaNO_2_ and kept in room temperature for 5 min. Subsequently, the tube was mixed with 0.15 ml of 10% AlCl_3_ and left to stand for 1 min. Finally, the reaction mixture was added 2 ml of 1 mol/L NaOH and 2.4 ml of distilled water and allowed to stand for 15 min in dark. The reaction mixture was centifuged for 5 min at 4,000 g and the absorbance was measured at 510 nm against an appropriate blank. A standard curve prepared using catechin was used to calculate the TFC as expressed as μmol catechin equivalents (CE) / g of dm.

### 3.7 Determination of proanthocyanidins content

Proanthocyanidins content was determined using the vanillin assay (Chandrasekara & Shahidi, 2010). Phenolic extracts (0.5 ml) in methanolic solution were mixed with 2.5 ml of 0.5% vanillin‐HCl reagent (0.5% vanillin (w/v) in 4% concentrated HCl in methanol) and was incubated for 20 min at room temperature. A separate sample was performed with 4% HCl in methanol as a blank. The absorbance was meassured at 500 nm, and the content of proanthocyanidins was expressed as μmol CE / g of dm.

### 3.8 Reducing power

The reducing power of samples was determined as explained by Chandrasekara and Shahidi (2010). Extract (0.5 ml) was mixed with 1.25 ml of phosphate buffer solution (0.2 mol/L, pH 6.6) and 1.25 ml of potassium ferricyanide in a centrifuge tube. The mixture was incubated for 20 min at 50°C and 1.25 ml of 10% TCA were added followed by centrifugation at 1750*g* for 10 min. The supernatant (1 ml) was transferred into a tube containing 1.25 ml of deionized water and 0.25 ml of 0.1% (w/v) FeCl_3_, and the absorbance values were read using a spectrophotometer at 700 nm. The standard curve was prepared using ascorbic acid. The results were expressed as μmol ascorbic acid equivalents (AAE) / g of dm.

### 3.9 DPPH radical scavenging activity

The DPPH radical scavenging activity of phenolic extract was determined using a spectrophotometric method (Lee, Emmy, Abbe Maleyki, & Amin, 2007). Briefly, 0.04 ml of methanolic extract (2 mg/ml) was added to 1.96 ml of methanolic DPPH (60 μM) solution. The mixture was vortexed and allowed to stand at room temperature in the dark for 20 min. The absorbance of the solutions was measured at 517 nm with the appropriate blank. The DPPH Radical Scavenging Activity (DRSA) was expressed as μmol trolox equivalents (TE) / g of dm.

### 3.10 Trolox equivalent antioxidant capacity

The trolox equivalent antioxidant capacity (TEAC) of soluble phenolic extracts of samples were determined by the method explained by Chandrasekara and Shahidi (2010). AAPH (2.5 mmol/L) was mixed with ABTS (100 mmol/L) in saline phosphate buffer (PBS) (pH 7.4, 0.15 mol/L NaCl) to prepare the ABTS• solution. The solution was kept in a water bath at 60°C for 16 min and the flask was covered by an aluminum foil to protect from light. Medium porosity filter papers were used to filter the prepared ABTS• solution before mixing with the extract. A separate blank was used to reduce the diminished absorbance of radical solution with time.

PBS solution was used to prepare millet phenolic extract (2 mg/ml) and further diluted to fit within the range of values (6.25–50 μmol/L) of the standard curve prepared using trolox.

To measure the TEAC, 40 μl of the extract was mixed with 1960 μl of the ABTS• solution. The absorbance of reaction mixture was measured at 734 nm immediately at the point of mixing (t_0_) and after 6 min (t_6_).

The absorbance reduction at 734 nm after 6 min of addition of trolox and extract was calculated using the following equation: ΔA trolox = (At0, trolox ‐ At6, trolox) ‐ (At0, blank ‐At6, blank), where ΔA is the reduction of absorbance and A the absorbance at a given time. TEAC values were expressed as μmol trolox equivalents (TE) / g of dm.

### 3.11 β *‐*carotene‐linoleate model system

Antioxidant activity of phenolic extracts was evaluated in a β‐carotene‐linoleate model system as explained by Chandrasekara and Shahidi (2010) with slight modifications. Briefly, 1 ml of β‐carotene (1 mg / ml) in chloroform was pipetted into a 100 ml round bottom flask. Chloroform was evaporated under vacuum using a rotary evaporator at room temperature. Subsequently, 40 mg of linoleic acid, 100 mg of Tween 80 emulsifier, and 100 ml of distilled water were added and the mixture was agitated vigorously to form an emulsion. The emulsion was freshly prepared for each experiment. Blank samples devoid of β‐carotene were prepared for the background subtraction. The extracts in methanol (200 μl) were added to the boiling tube containing 5 ml of emulsion and samples were kept in a water bath (50°C). The absorbance values were measured using a spectrophotometer at 470 nm wavelength immediately at zero time, 30 min and 60 min. Butylated hydroxyanisole (BHA) (200 ppm) in methanol was used as the reference standard. The antioxidant activity coefficient (AAC) after 60 min of incubation was calculated using the following equation: AAC = (Aa_(60)_–Ac_(60)_)/(Ac_(0)_–Ac_(60)_), where Aa_(60)_ and Ac_(60)_ are the absorbance values measured at 60 min for the sample and the control, respectively, and Ac _(0)_ is the absorbance value of the control at 0 min. The results were expressed as percentage of absorbance AAC / g of dm.

### 3.12 Ferrous ion chelating activity

Ferrous ion chelating ability of phenolic extracts was determined colorimetrically (Chandrasekara & Shahidi, 2010). The aliquot of 0.2 ml extract in distilled water was added to a solution of 2 mmol/L FeCl_2_ (0.025 ml) followed by addition of 0.2 ml of ferrozine (5 mmol/L) to initiate the reaction. The total volume of tube was adjusted up to 2 ml using distilled water and tubes were kept in room temperature for 10 min after vigorus shaking. The absorbance values were read at 562 nm. A separate control was prepared using distilled water in place of extract and blanks were arranged with added distilled water (1.8 ml) into 0.2 ml of sample for background subtraction. The inhibition percentage of Ferrozine‐ferrous ion complex formation was calculated by the following equation. Metal chelating effect (%) = [1‐(absorbance of the sample ‐ absorbance of the control)] 100. A standard curve was prepared using different EDTA concentrations (0.05–2 mmol/L) and the results were expressed as μmol EDTA equivalents / g of dm.

### 3.13 High‐performance liquid chromatoghraphy analysis

Predominantly available phenolic acid, ferulic acid of soluble and bound phenolic extracts of millet grains were identified and quantified using high‐performance liquid chromatoghraphy (HPLC) analysis. The reversed‐phase HPLC analysis was conducted by Shimadzu HPLC system (Shimadzu, SPD 20 A, Shimadzu Corporation, Kyoto, Japan) using Pinnacle^™^ II C‐18 column (4.6 × 150 mm, 5 μm, 110 Å, Restsk International, USA). The mobile phase was methanol/water (30:70 v/v). The flow rate was adjusted to 0.4 ml / min and the compounds were detected at 280 nm. All samples were filtered through a 0.45 μm syringe filter before injection. An external standard method was used to identify and quantify ferulic acid in millet samples. The results were expressed as μg / g of dm of millet samples.

### 3.14 Statistical analysis

All experiments were carried out in triplicates and data were reported as mean ± standard deviation. The differences of mean values among millet samples were determined by one‐way analysis of variance (ANOVA) followed by Tukey's honestly significant difference (HSD) multiple rank tests at *p* ≤ .05, significance level. Correlation analysis was performed between phenolic contents and antioxidant activity of soluble and bound extracts using Pearson correlations. All statistical analysis was performed by SPSS version 16 (SPSS Inc., Chicago, IL).

## 4 Results and Discussion

Millet samples used in this study consisted of different testa color and grain sizes. The weight of thousand grains of finger millet varieties *Ravi*,* Ravana*,* Oshada*, foxtail (PALL), and proso (ANUL) were 2.7, 3.3, 2.9, 1.1, and, 4.9 g, respectively. To the best of our knowledge, this study is the first to report on the phenolic contents and antioxidant activities of different millet varieties cultivated at a number of agro‐ecological zones in Sri Lanka. The proximate composition of dehulled millet grains are presented in Table 2.

**Table 2:** Proximate composition of millets

Variety	Moisture %	Crude fat content (g)	Ash Content(g)	Protein content(g)	Crude fiber content (g)
Finger millet					
MIRavi	10.0 ± 1.5	0.13 ± 0.02	2.72 ± 0.25	6.92 ± 0.53	3.41 ± 0.02
MIRav	12.4 ± 2.5	0.79 ± 0.20	1.83 ± 0.04	7.61 ± 0.22	3.04 ± 0.14
MIOsh	10.7 ± 0.1	0.84 ± 0.15	1.24 ± 0.17	7.17 ± 0.18	2.70 ± 0.01
MOL	11.3 ± 0.1	0.10 ± 0.00	1.24 ± 0.03	7.05 ± 0.55	3.43 ± 0.02
AML	11.4 ± 0.1	0.60 ± 0.06	0.73 ± 0.33	8.70 ± 0.25	3.59 ± 0.27
HAML	11.3 ± 0.2	0.27 ± 0.08	1.44 ± 0.30	7.59 ± 0.06	2.61 ± 0.10
WAL	11.4 ± 0.1	0.27 ± 0.08	1.69 ± 0.25	9.14 ± 0.23	3.45 ± 0.06
THAL	10.6 ± 0.6	0.59 ± 0.24	0.83 ± 0.40	7.42 ± 0.18	3.49 ± 0.03
ALUOsh	9.7 ± 0.01	0.59 ± 0.06	2.15 ± 0.30	7.54 ± 0.48	3.00 ± 0.33
NIKOsh	10.1 ± 0.1	0.34 ± 0.19	1.65 ± 0.11	7.29 ± 0.24	2.22 ± 0.09
BATRav	11.3 ± 3.2	0.48 ± 0.05	2.20 ± 0.08	8.03 ± 0.25	3.01 ± 0.17
Foxtail millet					
PALL	10.2 ± 0.9	4.50 ± 0.35	2.17 ± 0.36	9.50 ± 0.49	1.52 ± 0.19
HAML	11.6 ± 1.0	3.84 ± 0.01	1.54 ± 0.29	9.93 ± 0.28	2.35 ± 0.32
MOL	11.4 ± 0.4	4.58 ± 0.05	1.69 ± 0.35	11.01 ± 0.19	1.77 ± 0.15
JAFL	10.1 ± 1.0	4.23 ± 0.08	1.83 ± 0.03	10.23 ± 0.21	2.38 ± 0.39
Proso millet					
ANUL	12.6 ± 0.3	3.32 ± 0.30	2.26 ± 0.24	10.70 ± 0.35	2.35 ± 0.11
HAML	13.0 ± 0.6	3.09 ± 0.20	2.17 ± 0.33	9.37 ± 0.59	2.30 ± 0.08

Nutrient composition per 100 g of edible portion.

### 4.1 Total phenolic content

The Folin‐Ciocalteu's assay used to determine the TPC is based on the reducing ability of hydroxyl groups attached to phenolic compounds of the extracts. In this study, the TPC of soluble phenolic extracts of whole grain millets, dehulled grains, and their counterpart hulls ranged from 4.3 to 52.3, 0.4 to 32.5, and 10.9 to 44.4 μmol FAE / g dm, respectively (Table 3). In agreement with the previous studies, TPC of hulls were higher compared to those of dehulled and whole grains of studied millet samples (Chandrasekara, Naczk, & Shahidi, 2012; Chandrasekara & Shahidi, 2011; Varsha et al., 2009).

**Table 3:** Total phenolic content (TPC) and total flavonoid content (TFC) of soluble phenolic extract of millets

Sample	TPC μmol of ferulic acid equiv/g of dry matter			TFC μmol of catechin equiv/g of dry matter		
	Whole grains	Dehulled grains	Hull	Whole grains	Dehulled grains	Hull
Finger millet						
MIRavi	15.1 ± 0.2^a1^	17.8 ± 0.2^a2^	39.9 ± 0.6^a3^	8.0 ± 2.7^a1^	8.3 ± 1.1^a2^	9.5 ± 0.2^a2^
MIRav	18.0 ± 0.2^b1^	22.3 ± 0.1^b2^	38.9 ± 0.9^a3^	8.6 ± 0.2^ab1^	8.3 ± 1.1^a1^	13.2 ± 3.2^b2^
MIOsh	18.4 ± 0.2^c1^	32.5 ± 0.4^c2^	33.8 ± 0.3^b3^	12.5 ± 0.1^c1^	11.0 ± 1.3^b1^	12.9 ± 2.8^b1^
MOL	27.0 ± 0.3^d1^	20.6 ± 0.2^d2^	40.0 ± 0.1^a3^	8.0 ± 0.1^a1^	8.5 ± 1.2^a1^	22.1 ± 3.1^c2^
AML	52.3 ± 0.5^e1^	21.8 ± 0.4^b2^	14.6 ± 0.4^c3^	7.8 ± 0.1^a1^	10.5 ± 1.1^b2^	13.7 ± 0.7^b3^
HAML	15.5 ± 0.1^f1^	23.1 ± 0.6^e2^	36.9 ± 1.5^d3^	8.1 ± 0.6^a1^	8.5 ± 0.7^a1^	10.4 ± 0.4^a2^
WAL	18.8 ± 0.1 ^g1^	20.5 ± 0.1^d2^	39.3 ± 1.3^a3^	2.5 ± 0.1^d1^	3.1 ± 0.5^c1^	14.9 ± 1.9^b2^
THAL	22.7 ± 0.3 ^h1^	13.3 ± 0.3^f2^	44.4 ± 2.8^e3^	8.9 ± 0.2^a1^	6.4 ± 0.8^d2^	13.1 ± 1.9^b2^
ALUOsh	20.5 ± 0.1^i1^	14.3 ± 0.1 ^g2^	43.5 ± 0.2^f3^	9.4 ± 0.2^a1^	1.1 ± 0.2^e2^	14.8 ± 0.3^b3^
NIKOsh	19.5 ± 0.1^j1^	17.0 ± 0.3 ^h1^	24.9 ± 2.7 ^g2^	9.6 ± 0.2^b1^	5.6 ± 0.5^d2^	10.1 ± 1.0^a1^
BATRav	23.4 ± 0.1^k1^	23.2 ± 0.6^e1^	25.3 ± 0.7 ^g2^	8.6 ± 0.4^a1^	6.8 ± 0.6^d1^	21.3 ± 2.7^c2^
Foxtail millet						
PALL	6.3 ± 0.3^a1^	1.4 ± 0.1^a2^	10.9 ± 0.8^a3^	1.4 ± 0.2^a1^	1.0 ± 0.1^a2^	3.0 ± 0.2^a3^
HAML	5.6 ± 0.9^a1^	2.0 ± 0.7^a2^	17.6 ± 1.4^b3^	3.5 ± 0.3^a1^	2.0 ± 0.1^a2^	6.6 ± 0.2^a3^
MOL	9.1 ± 0.3^b1^	1.7 ± 0.2^a2^	16.6 ± 0.4^b3^	2.5 ± 0.2^a1^	1.0 ± 0.1^a2^	4.0 ± 0.1^b3^
JAFL	6.0 ± 0.2^a1^	0.4 ± 0.1^b2^	28.3 ± 1.5^c3^	1.4 ± 0.2^a1^	1.2 ± 0.1^a1^	4.1 ± 0.2^b2^
Proso millet						
ANUL	4.3^ ^± 0.2^a1^	1.2^ ^± 0.1^a2^	14.9^ ^± 0.8^a3^	1.3 ± 0.1^a1^	1.2 ± 0.3^a2^	2.4 ± 0.1^a3^
HAML	10.4^ ^± 0.3^b1^	5.5 ± 0.2^b2^	35.8 ± 2.2^b3^	2.1 ± 0.1^b1^	1.4 ± 0.4^b2^	13.1 ± 3.2^b3^

Same letters in each column and same numbers in each raw for each test are not significantly different (*p* > .05).

Finger, proso, and foxtail millets belong to three species, namely *Eleusine coracana, Panicum miliaceum*, and *Setaria italica,* respectively. It was reported that millets with dark color pigmented testa and pericarp showed higher phenolic content of soluble phenolic fractions than those with light color such as white or yellow testa (Chandrasekara & Shahidi, 2010). In agreement, soluble phenolic extracts of finger millets in this study had more TPC compared to those of foxtail and proso millets.

The varietal effect on TPC of millet extracts within the species is clearly demonstrated in this study. A significant difference of TPC among finger millet varieties, cultivated in the same location was observed in this study (Table 3). Earlier, considerable differences in 0.19 to 3.37% (catechin equivalents) of TPC among 85 Indian finger millet varieties were reported (Shankara, 1991). Varietal variations in respect to the TPC of finger millets have been reported in other studies too (Chethan & Malleshi, 2007).

The differences in the TPC of soluble extracts of millet grains due to cultivated locations were presented in Table 3. Sri Lanka is a country with a heterogeneous agro‐ecological environment. Based on rainfall distribution, there are traditionally three climatic zones, namely wet zone, dry zone, and intermediate zone in Sri Lanka. In this study, millet samples were collected from agro‐ecological regions in dry and intermediate zones (Table 1). According to the results, the millet samples obtained from agro‐ecological regions in dry zone had more TPC compared to those from intermediate zone (Table 3). The dry zone receives a mean annual rainfall of less than 1750 mm with a distinct dry season from May to September and the intermediate zone receives a mean annual rainfall between 1750 and 2500 mm with a short and a less prominent dry season .

Environmental factors such as sun exposure, soil type, and rainfall have an effect on phenolic content of plants (Manach et al., 2004). Low temperature may increase the production of phenolics by enhancing synthesis of phenylalanine ammonia lyase (PAL) in plants, while high altitude and long sunlight exposure with high UV radiation positively affect the synthesis of phenolic compounds (Kishore, Ranjan, Pandey, & Gupta, 2010).

The TPC of bound phenolic extracts of representative millet samples were shown in Table 4. Bound phenolic extracts of millet whole grains and hulls contain more TPC compared to that of soluble counterparts. Soluble phenolic extracts of millet dehulled grains contain more TPC compared to those of bound counterparts. In a previous study, a low TPC of bound phenolic extracts of the whole grain of finger millet (variety *Ravi*) compared to their soluble counterparts was reported (Chandrasekara & Shahidi, 2010). In this study, the bound phenolic extracts of whole grains and hulls of foxtail millets had higher TPC compared to their soluble counterparts. Chandrasekara and Shahidi (2010) also reported high TPC in bound phenolic extracts of foxtail millet whole grains.

**Table 4:** Total phenolic content (TPC), total flavonoid content (TFC), 2, 2‐diphenyl‐1‐picrylhydrazyl (DPPH) radical scavenging ability, and reducing power (RP) of bound phenolic extracts of representative millet samples

** **	TPC μmol of ferulic acid equiv/g of dry matter			TFC μmol of Catechin equiv/g of dry matter		
	Whole grains	Dehulled grains	Hull	Whole grains	Dehulled grains	Hull
Finger millet						
MIRavi	26.8 ± 0.2^a1^	0.7 ± 0.1^a2^	59.0 ± 2.1^a3^	0.03 ± 0.01^a1^	0.06 ± 0.02^a1^	0.27 ± 0.05^a2^
MIRav	27.5 ± 2.2^a1^	0.7 ± 0.2^a2^	49.2 ± 2.3^b3^	0.07 ± 0.04^ab1^	0.04 ± 0.01^a1^	0.21 ± 0.04^a2^
MIOsh	29.5 ± 0.7^b1^	2.2 ± 0.1^b2^	56.7 ± 1.7^a3^	0.07 ± 0.04^ab1^	0.05 ± 0.02^a1^	0.62 ± 0.11^c2^
Foxtail millet						
HAML	14.7 ± 0.8^c1^	1.4 ± 0.2^a2^	34.1 ± 0.4^d3^	0.26 ± 0.04^c1^	0.95 ± 1.07^b2^	0.20 ± 0.03^a1^
MOL	38.1 ± 0.1^d1^	0.7 ± 0.1^a2^	39.9 ± 3.9^c3^	0.11 ± 0.03^b1^	0.02 ± 0.01^a2^	0.07 ± 0.01^d1^
JAFL	32.4 ± 1.9^e1^	2.9 ± 0.9^c2^	33.3 ± 1.3^d3^	0.09 ± 0.03^b1^	0.01 ± 0.00^a2^	0.15 ± 0.05^b1^
Proso millet						
ANUL	3.4 ± 0.1^f1^	5.1 ± 1.2^d1^	25.6 ± 0.8^e2^	0.02 ± 0.01^a1^	0.06 ± 0.01^a2^	0.06 ± 0.02^d2^
HAML	4.2 ± 0.1^f1^	1.5 ± 0.3^a1^	25.9 ± 2.1^e2^	0.03 ± 0.01^a1^	0.06 ± 0.02^a2^	0.12 ± 0.03^d3^

	DPPH μmol of trolox equiv/g of dry matter			RP μmol of ascorbic acid equiv/g of dry matter		
	Whole grains	Dehulled grains	Hull	Whole grains	Dehulled grains	Hull
Finger millet						
MIRavi	0.45 ± 0.03^a1^	0.49 ± 0.03^a1^	2.48 ± 0.08^a2^	3.82 ± 0.28^a1^	2.60 ± 0.33^a2^	10.98 ± 0.36^a3^
MIRav	1.09 ± 0.03^b1^	0.94 ± 0.05^b2^	1.82 ± 0.07^b3^	6.19 ± 0.13^b1^	4.67 ± 0.13^b2^	12.70 ± 1.29^b3^
MIOsh	0.82 ± 0.02^c1^	0.36 ± 0.04^c2^	2.40 ± 0.04^a3^	6.48 ± 0.34^b1^	3.37 ± 0.70^c2^	20.59 ± 1.77^c3^
Foxtail millet						
HAML	0.82 ± 0.01^c1^	0.23 ± 0.01^d2^	0.85 ± 0.01^c1^	3.86 ± 0.32^c1^	2.37 ± 0.25^de2^	8.14 ± 0.77^a3^
MOL	2.35 ± 0.02^d1^	0.72 ± 0.06^e2^	1.91 ± 0.19^b3^	6.21 ± 0.20^d1^	6.08 ± 0.35^b1^	1.49 ± 0.42^b2^
JAFL	2.01 ± 0.07^e1^	0.51 ± 0.05^a2^	0.85 ± 0.08^c3^	9.17 ± 0.79^e1^	1.90 ± 0.11^e2^	11.19 ± 0.63^b3^
Proso millet						
ANUL	0.35 ± 0.02^f1^	0.46 ± 0.08^a2^	0.69 ± 0.04^d3^	15.80 ± 0.27^a1^	4.67 ± 0.23^d2^	13.19 ± 0.30^d3^
HAML	0.72 ± 0.01 ^g1^	0.31 ± 0.04^c2^	0.75 ± 0.02^d1^	7.99 ± 0.94^b1^	1.72 ± 0.28^f2^	13.33 ± 1.10^e3^

Same letters in each column and same numbers in each raw for different tests are not significantly different (>0.05).

The trend of TPC of soluble and bound phenolic extracts of proso millets were different between the two cultivated locations in this study. According to Zhang, Ruihai, and Wei (2014), the TPC of bound phenolic extracts of dehulled proso millets were significantly higher than those of free phenolics. Further, the TPC of free phenolics extracts of three different varieties of proso millet ranged from 27.48 to 151.14 mg gallic acid equiv (GAE)/100 g dm. In addition, the bound phenolic content ranged from 55.95 (Gumi20) to 305.81 (Mi2504‐6) mg GAE/100 g dm (Zhang et al., 2014).

### 4.2 Total flavonoid content

Flavonoids are polyphenolic compounds comprising of 15 carbons, with two aromatic rings connected by a 3‐carbon bridge. According to the modifications of the central C‐ring, they can be divided into different structural classes such as flavonols, flavones, flavan‐3‐ols, flavanones, isoflavones, and anthocyanidins. Determination of total flavonoid content is based on the chelating ability of flavonoids with aluminum (III). Flavonoids form a pink‐colored complex with aluminum (III) through the 4‐keto and neighboring hydroxyl groups or through adjacent hydroxyl groups in the B ring (Kim, Jeong, & Lee, 2003).

Finger millet showed the highest TFC of soluble extracts followed by foxtail millets and proso millets (Table 3). An earlier study also showed that finger millets had higher TFC compared to foxtail and proso millets (Chandrasekara & Shahidi, 2010, 2011). Soluble extracts of millet hull had more TFC compared to those of dehulled grains and whole grains. The TFC of bound extracts of whole grains, dehulled grains and hulls of millet samples ranged from 0.03 to 0.26, 0.01 to 0.95, and 0.06 to 0.62 μmoles of CE/g dm, respectively (Table 4). Earlier, a higher TFC of millet grains in soluble phenolic extracts was reported compared to those of bound millet grains (Chandrasekara & Shahidi, 2010). TFC was significantly influenced by the variety and cultivated locations of millets as shown in the present work (Table 3). Ju‐Sung, Tae, and Myong‐Jo (2010) also showed that the TFC of five cultivars of whole grains of proso millet ranged from 3.4 to 11.5 mg quercertin equivalents (QE) /g of sample. Further, the TFC of eight cultivars of whole grains of foxtail millet ranged from 4.0 to 8.1 mg QE/g of sample. The changes of the TFC of millets in different cultivated locations may be attributed to the environmental conditions affected during the plant growth.

### 4.3 Proanthocyanidin content

**Figure 1:**
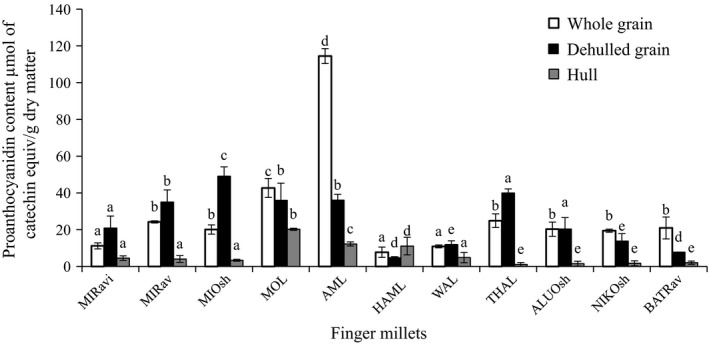
Proanthocyanidin content (PC) of soluble phenolic extracts of finger millets. (MIRavi‐variety *Ravi*, MIRav‐variety *Ravana*, MIOsh‐variety *Oshada* from Mahailluppallama, AML‐local variety from Ampara, HAML‐local variety from Hambanthota, WAL‐local varity from Wariyapola, THAL‐Local variety from Thabuththegama, ALUOsh variety *Oshada* from Aluththarama, NIKOsh‐variety *Oshada* from Nikaweratiya, BATRav variety *Ravi* from Bataatha)**.** Same letters in each category (whole grain, dehulled grain, hull) are not significantly different (*p* ****> .05)

Proanthocyanidin or condensed tannins are oligomers or polymers of flavan‐3‐ ol units and they are synthesized via the phenyl propanoid pathways. Figure 1 shows the proanthocyanidins content of finger millets. The highest proanthocyanidin content (PC) was found in whole and dehulled grains compared to those of hulls. Siwela, Taylor, De Milliano, and Duodu (2007) did provide evidence to show that tannins in finger millet are located in the testa layer.

There is a significant diversity among the PC of three different finger millet varieties cultivated in the same location and finger millets cultivated in different locations. The highest PC was found among the finger millets cultivated agro‐ecological regions of DL 1, 2, and 5 in the dry zone compared to those of intermediate zone in Sri Lanka (Fig. 1).

The PC of foxtail millets and proso millets were not detectable. In a previous study, the highest PC of 311.28 ± 3.0 μmol CE/g of defatted meal for finger millet local variety was reported followed by finger millet (variety *Ravi*), foxtail, little, pearl, and proso millets (Chandrasekara & Shahidi, 2010). They further reported a low amount of PC in proso millets and foxtail millets grains. Condensed tannins are biologically active and when present in sufficient quantities, may lower the nutritional value of food and biological availability of proteins and minerals. However, they possess anti‐inflammatory, antiviral, antibacterial, and antioxidant properties (Fei, Qiu, Ying, & Chang, 2008). In this study, the PC of whole grain finger millets were positively associated with TPC (r = 0.985; *p* < .001). This was in agreement with Siwela et al. (2007) who also reported a high significant positive correlation between total phenolics and condensed tannin contents (r = .927; *p* < .001) of finger millet, indicating the high contribution of condensed tannins to the TPC of finger millets.

### 4.4 Reducing power

The reducing power (RP) assay is a method to determine the total antioxidant power of a plant extract. The method is based on the ability of compounds to donate electrons to reduce the ferricyanide to ferrocyanide. Table 5 presents the RP of whole grains, dehulled grains, and hulls of soluble phenolic extracts and they ranged from 3.3 to 23.9, 1.3 to 18.0, and 5.7 to 60.4 μmol of AAE/ g dm, respectively. In another study, several millet extracts (finger millet, kodo millet, proso millet, pearl millet, little millet, and foxtail millet) consisted of considerable RP and, finger millet showed the highest RP, whereas proso millet had the lowest (Chandrasekara & Shahidi, 2010). In agreement, this study also showed the highest RP value in soluble extracts of finger millets. The RP values of bound phenolic extracts of millet samples were shown in Table 4.

**Table 5:** Reducing Power (RP), Trolox Equivalents Antioxidant Capacity (TEAC), and percentage of ß caroteine oxidation inhibition of soluble extracts of millets

	RP μmol of ascorbic acid equiv/g of dry matter			TEAC μmol of trolox equiv/g of dry matter			Percentage of ß caroteine oxidation inhibition		
	Whole grains	Dehulled grains	Hull	Whole grains	Dehulled grains	Hull	Whole grains	Dehulled grains	Hull
Finger millet									
MIRavi	14.7 ± 0.1^a1^	7.0 ± 0.6^a2^	52.1 ± 0.7^a3^	4.6 ± 0.4^a1^	3.8 ± 0.2^a2^	4.8 ± 0.8^a1^	21.0 ± 1.2^a1^	25.7 ± 3.6^a1^	31.8 ± 4.7^a2^
MIRav	18.4 ± 0.1^b1^	13.3 ± 0.2^b2^	36.2 ± 1.0^b3^	2.4 ± 0.5^b1^	2.4 ± 0.0^b1^	2.8 ± 0.6^b1^	24.8 ± 0.3^ab1^	11.5 ± 2.0^b2^	34.4 ± 3.3^a3^
MIOsh	17.1 ± 0.1^c1^	12.4 ± 0.2^c2^	33.4 ± 1.3^c3^	3.7 ± 0.3^c1^	3.1 ± 0.3^c2^	4.9 ± 0.9^a1^	6.7 ± 0.2^c1^	36.1 ± 4.8^c2^	41.2 ± 3.0^b2^
MOL	23.9 ± 0.2^d1^	12.9 ± 0.9^c2^	50.1 ± 1.4^d3^	2.1 ± 0.3^b1^	1.7 ± 0.1^d2^	4.6 ± 1.0^a2^	38.6 ± 0.3^d1^	53.7 ± 1.4^d2^	60.4 ± 3.6^c3^
AML	21.4 ± 0.3^e1^	18.0 ± 0.3^d2^	16.5 ± 0.2^e3^	4.8 ± 0.0^a1^	3.1 ± 0.4^c2^	6.1 ± 0.2^c2^	50.5 ± 0.2^e1^	63.1 ± 2.9^e2^	91.7± 0.4^d3^
HAML	16.9 ± 0.1^c1^	8.0 ± 0.1^e2^	39.8 ± 1.6^f3^	6.6 ± 0.6^d1^	3.0 ± 0.2^c2^	8.9 ± 0.2^d3^	74.7 ± 7.5^f1^	31.5 ± 5.1^c2^	76.9 ± 2.3^e1^
WAL	21.5 ± 0.1^e1^	3.8 ± 0.1^f2^	22.8 ± 1.2^ g3^	5.9 ± 0.3^e1^	5.9 ± 0.2^e2^	6.5 ± 0.0^c3^	68.4 ± 0.2^ g1^	35.8 ± 2.4^c2^	72.3 ± 1.9^e1^
THAL	8.6 ± 0.1^f1^	12.3 ± 0.1^c2^	44.8 ± 0.1^ h3^	3.2 ± 0.0^c1^	1.7 ± 0.2^d2^	4.5 ± 0.6^a3^	35.2 ± 1.1^ h1^	43.7 ± 7.1^e2^	50.1 ± 2.3^f2^
ALUOsh	11.1 ± 0.1 ^g1^	2.2 ± 0.1^ g2^	46.3 ± 0.4^ h^	3.1 ± 0.6^c1^	3.6 ± 0.3^a2^	4.9 ± 0.2^a2^	36.1 ± 0.4^ h1^	46.8 ± 11.6^e2^	54.1 ± 1.3^f2^
NIKOsh	13.3 ± 0.1 ^h1^	5.5 ± 0.2^ h2^	45.1 ± 0.1^ h3^	4.3 ± 0.5^ac1^	3.6 ± 0.5^a1^	4.4 ± 1.0^a1^	27.6 ± 0.3^b1^	25.2 ± 4.2^a1^	33.9 ± 3.6^a2^
BATRav	20.9 ± 0.1^i1^	7.5 ± 0.5^ae2^	60.4 ± 0.5^i3^	2.2 ± 0.7^b1^	1.0 ± 0.2^f2^	3.4 ± 0.0^b3^	23.7 ± 0.6^a1^	11.5 ± 3.1^b2^	33.9 ± 2.9^a3^
Foxtail millet									
PALL	3.3 ± 0.3^a1^	1.3 ± 0.1^a2^	7.4 ± 0.2^a3^	0.8 ± 0.2^a1^	0.7 ± 0.1^a1^	2.0 ± 1.0^a2^	69.8 ± 0.3^a1^	75.6 ± 0.4^a2^	79.4 ± 0.5^a3^
HAML	4.5 ± 0.3^b1^	2.6 ± 0.5^b2^	11.0 ± 0.4^b3^	3.7 ± 0.3^b1^	1.4 ± 0.1^b2^	3.8 ± 0.2^b1^	67.8 ± 0.7^b1^	29.5 ± 0.2^b2^	69.3 ± 0.6^b3^
MOL	4.1 ± 0.1^b1^	1.4 ± 0.3^c2^	5.7 ± 0.1^c3^	5.2 ± 0.2^c1^	2.3 ± 0.2^c2^	3.3 ± 0.4^c3^	63.4 ± 0.3^c1^	15.6 ± 0.5^c2^	73.3 ± 0.4^c3^
JAFL	2.6 ± 0.4^c1^	2.4 ± 0.4^c2^	5.9 ± 0.1^c1^	2.3 ± 0.3^d1^	1.6 ± 0.1^b2^	2.3 ± 0.1^a1^	51.6 ± 0.8^d1^	30.5 ± 0.8^d2^	50.6 ± 0.5^d1^
Proso millet									
ANUL	4.1 ± 0.4^a1^	1.7 ± 0.6^a2^	8.7 ± 0.1^a3^	0.3 ± 0.1^a1^	0.2 ± 0.1^a1^	3.0 ± 0.4^a2^	51.6 ± 1.1^a1^	45.3 ± 0.6^a2^	65.8 ± 0.6^a3^
HAML	5.6 ± 0.6^b1^	3.8 ± 0.1^b2^	18.1 ± 0.9^b3^	2.5 ± 0.09^b1^	1.4 ± 0.1^b2^	4.0 ± 0.4^b3^	34.7 ± 0.9^b1^	57.9 ± 0.5^b2^	48.7 ± 0.9^b3^

Same letters in each column and same numbers in each raw are not significantly different (*p* > .05).

Antioxidant activity as measured by RP values of soluble and bound phenolic extracts are significantly different among morphological parts of the millet seeds. The RP of soluble and bound phenolic extracts of studied millet hulls were higher than the counterparts of dehulled grains and whole grains, and the process of dehulling reduced the RP values of whole millet grains. It has been shown that the RP of finger millet seed coat extract was higher (*p* < .01) than that of whole flour extract (Varsha et al., 2009). Ju‐Sung et al. (2010) established a positive linear correlation between TPC and RP of sorghum, foxtail millet, and proso millet (r = .985). This study also indicated a positive association between TPC and RP of finger millets (r = .367, *p* < .001).

### 4.5 DPPH radical scavenging ability

DPPH is a synthetic stable free radical which can be scavenged by the donated hydrogen from the antioxidative compound. The DPPH• radical displays an intense UV‐VIS absorption spectrum. The free radicals left, after reacting with phenolic compounds present in the samples were measured using the UV‐VIS spectrophotometer.

The DRSA of bound and soluble phenolic extracts of whole grains, dehulled grains, and hulls of millets were presented in Table 4 and Table 6, respectively. Finger millets showed the highest DRSA in their soluble extracts compared to those of foxtail and proso millets. Finger millet dehulled grains had higher DRSA compared to those counterparts of whole grains and hulls. The DRSA of soluble extracts of dehulled finger millet grains were associated with PC (r = .963; *p* < .001) and TFC (r = .771; *p* < .001). In a previous study, high DRSA, has been documented that could be due to high content of phenolic compounds such as tannin and flavonoids in finger millets (Yokozawa et al., 1998).

**Table 6:** 2, 2‐diphenyl‐1‐picrylhydrazyl (DPPH) radical scavenging ability and ferrous ion chelating ability of soluble phenolic extracts of millets

	DPPH μmol of trolox equiv/g of dry matter			Fe chelating ability μmol of EDTA equiv/g dry matter		
	Whole grains	Dehulled grains	Hull	Whole grains	Dehulled grains	Hull
Finger millet						
MIRavi	7.9 ± 0.2^a1^	12.5 ± 0.3^a2^	8.6 ± 1.0^a3^	4.7 ± 0.7^a1^	8.2 ± 0.4^a2^	3.2 ± 0.1^a3^
MIRav	8.8 ± 0.2^a1^	15.9 ± 0.7^b2^	5.9 ± 1.0^b3^	4.3 ± 0.2^a1^	6.8 ± 0.1^b2^	0.4 ± 0.1^b3^
MIOsh	10.3 ± 0.7^b1^	21.0 ± 0.8^c2^	3.4 ± 0.5^c3^	3.5 ± 0.1^b1^	8.8 ± 0.1^c2^	1.8 ± 0.4^c3^
MOL	10.8 ± 1.7^b1^	15.7 ± 0.2^b2^	7.2 ± 0.8^d3^	7.5 ± 0.1^c1^	7.2 ± 0.1^d2^	1.6 ± 0.1^d3^
AML	24.7 ± 0.9^c1^	26.4 ± 0.7^c1^	8.1 ± 0.3^ad2^	6.1 ± 0.2^d1^	7.4± 0.1^d2^	0.3 ± 0.0^b3^
HAML	5.7 ± 0.1^d1^	14.1 ± 0.3^d2^	0.8 ± 0.8^e3^	3.3 ± 0.4^be1^	7.9 ± 0.2^e2^	2.3 ± 0.1^e3^
WAL	7.7 ± 1.0^a1^	11.9 ± 0.8^e2^	5.3 ± 0.5^b3^	5.2± 0.6^a1^	5.6 ± 0.1^f1^	3.1 ± 0.2^a3^
THAL	11.9 ± 0.9^b1^	18.5 ± 0.9^f2^	10.1 ± 1.1^f1^	5.8 ± 0.1^d1^	8.2 ± 0.1^a2^	2.6 ± 0.2^f3^
ALUOsh	6.8 ± 0.3^ad1^	7.6 ± 1.1^ g1^	4.1 ± 0.1^cb2^	5.1 ± 1.3^a1^	5.1 ± 0.01^ g1^	1.2 ± 0.1^ g3^
NIKOsh	8.6 ± 0.4^a1^	12.8 ± 1.3^a2^	8.0 ± 0.9^ad1^	2.5 ± 0.5^e1^	6.1 ± 0.1^ h2^	3.8 ± 0.1^ h3^
BATRav	10.4 ± 1.1^b1^	18.3 ± 0.9^f2^	5.1 ± 0.9^b3^	3.7 ± 0.7^ab1^	10.0 ± 0.1^i2^	3.4 ± 0.1^a3^
Foxtail millet						
PALL	2.1 ± 0.1^a1^	1.2 ± 0.1^a2^	2.2 ± 0.3^a1^	1.3 ± 0.1^a1^	2.6 ± 0.1^a2^	1.1 ± 0.01^a1^
HAML	1.7 ± 0.01^a1^	0.9 ± 0.4^b2^	5.0 ± 0.4^b3^	0.2 ± 0.1^b1^	5.2 ± 0.1^b2^	0.4 ± 0.01^b3^
MOL	4.0 ± 0.9^b1^	1.1 ± 0.1^ab2^	1.8 ± 0.1^a2^	0.2 ± 0.01^b1^	3.6 ± 0.1^c2^	0.4 ± 0.1^c3^
JAFL	0.8 ± 0.1^c1^	0.3 ± 0.1^c2^	4.7 ± 0.4^b3^	0.4 ± 0.01^c1^	5.9 ± 0.1^d2^	0.7 ± 0.01^d3^
Proso millet						
ANUL	1.5 ± 0.2^a1^	1.8± 0.2^a1^	9.6 ± 0.9^a2^	0.2 ± 0.0^a1^	0.1 ± 0.01^a2^	0.1 ± 0.01^a2^
HAML	0.4 ± 0.2^b1^	0.6 ± 0.3^b1^	3.3 ± 0.7^b2^	0.1 ± 0.0^b1^	0.5 ± 0.01^b2^	0.1 ± 0.1^a3^

Same letters in each column and same numbers in each raw for each test are not significantly different (*p* > .05).

Variety *Oshada* of finger millet showed 68% and 32% higher DRSA of soluble phenolic extracts of dehulled grains than those of *Ravi* and *Ravana* varieties, respectively. Results of present study clearly demonstrated the differences in DRSA among finger millet varieties. A similar finding was reported for brown or red variety of finger millet, which were commonly available, with higher DRSA of 94% than those of white varieties which shown only 4% (Hegde & Chandra, 2005).

In this study, DRSA of millets were significantly different among the cultivated locations. Recently, it was shown that the DRSA of proso millet is affected by the growing environment (Kejariwal & Mehra, 2014). Authors further revealed that the proso millet organically grown had higher percentage of DRSA compared to the conventionally grown proso millet. Mpofu, Sapirstein, and Beta (2006) demonstrated that antioxidant activities of Canadian wheat cultivars were affected by the environment where they were grown. It was shown that environmental factors such as temperature, sunlight exposure, and altitude might be the significant factors affecting some antioxidant properties for tartary buckwheat flour (Xu‐Dan et al., 2011). These results added more evidence to the results obtained in this study.

### 4.6 TEAC

TEAC assay is based on the scavenging ability of antioxidants to the long‐life radical anion ABTS•. In this assay, ABTS is oxidized by AAPH to its radical cation, ABTS•, which is having intense blue‐green color. The antioxidant ability was measured as the ability of test compounds to decrease the color reacting directly with the ABTS• radical. Results of test compounds are expressed relative to trolox, water soluble analog of α‐tocopherol.

The TEAC of soluble phenolic extracts of whole grains, dehulled grains, and hulls of millets were presented in the Table 5. The TEAC of soluble extracts was in the order of finger > foxtail > proso millets. The soluble phenolic extracts of hulls had higher TEAC compared to the whole grains and dehulled grains of studied millet samples. The TEAC of bound phenolic extracts of whole grains, dehulled grains, and hulls of representative millet samples ranged from 2.0 to 2.7, 0.2 to 1.5, and 1.5 to 4.5 μmoles of TE/g of dm, respectively (Table 7). Bound phenolic extracts of finger millet exhibited a lower TEAC than their soluble counterparts. Bound extracts of foxtail and proso millet showed higher TEAC than their soluble counterparts. The above trend of TEAC among different millet varieties used in the present work is in agreement with the previous studies reported for millet. Chandrasekara and Shahidi (2010) showed that bound phenolic extracts of proso millet and foxtail millets showed a higher TEAC compared to that of soluble. Xu‐Dan et al. (2011) also demonstrated a greater ABTS radical scavenging ability of free phenolic compounds than that of bound of Tartary buckwheat. Further, authors reported that free phenolic extracts were the major contributors of the total radical scavenging capacity of Tartary buckwheat (Xu‐Dan et al., 2011).

**Table 7:** Trolox eequivalent antioxidant capacity (TEAC; μmol of trolox equiv/g of dry matter), ferrous ion chelating ability (μmol of EDTA equiv/g of dry matter), and percentage of β carotene oxidation inhibition of bound phenolic extracts of millet

	Whole grains	Dehulled grains	Hull
TEAC			
Finger millet			
MIRavi	2.60 ± 0.01^a1^	0.40 ± 0.02^a2^	4.53 ± 0.02^a3^
MIRav	2.50 ± 0.02^b1^	0.16 ± 0.02^b2^	2.73 ± 0.02^b3^
MIOsh	2.68 ± 0.03^c1^	0.70 ± 0.03^c2^	1.49 ± 0.01^c3^
Foxtail millet			
HAML	2.00 ± 0.02^f1^	1.44 ± 0.01^d2^	2.27 ± 0.01^e3^
MOL	2.02 ± 0.01^f1^	1.45 ± 0.01^d2^	2.26 ± 0.01^e3^
JAFL	1.77 ± 0.04^d1^	1.33 ± 0.00^e2^	1.46 ± 0.01^f3^
Proso millet			
ANUL	1.26 ± 0.01^e1^	1.04 ± 0.02^f2^	1.49 ± 0.01^c3^
HAML	2.01 ± 0.01^f1^	1.48 ± 0.03^d2^	1.71 ± 0.01^d3^
Fe chelating ability			
Finger millet			
MIRavi	1.43 ± 0.09^a1^	1.49 ± 0.01^a1^	1.67 ± 0.04^a2^
MIRav	1.47 ± 0.01^ab1^	1.46 ± 0.05^b1^	1.49 ± 0.01^b1^
MIOsh	1.50 ± 0.01^b1^	1.42 ± 0.01^c2^	2.04 ± 0.02^c3^
Foxtail millet			
HAML	1.23 ± 0.01^c1^	1.34 ± 0.01^d2^	0.83 ± 0.01^d3^
MOL	1.35 ± 0.01^d1^	1.06 ± 0.01^e2^	0.87 ± 0.01^e3^
JAFL	1.36 ± 0.01^d1^	1.35 ± 0.01^f1^	0.94 ± 0.02^f2^
Proso millet			
ANUL	1.38 ± 0.01^d1^	1.26 ± 0.01^g2^	1.14 ± 0.02^g3^
HAML	1.36 ± 0.01^d1^	1.23 ± 0.01^h2^	1.11 ± 0.02^g3^
Percentage of ß carotene oxidation inhibition			
Finger millet			
MIRavi	61.41 ± 1.93^a1^	37.88 ± 0.63^a2^	55.52 ± 0.76^a3^
MIRav	40.27 ± 0.79^b1^	40.32 ± 0.52^b1^	40.46 ± 0.75^b1^
MIOsh	66.24 ± 0.72^c1^	32.76 ± 0.65^c2^	51.36 ± 1.41^c3^
Foxtail millet			
HAML	43.52 ± 0.58^b1^	28.79 ± 0.30^f2^	43.52 ± 0.58^b1^
MOL	72.02 ± 0.66^e1^	38.26 ± 1.20^c2^	51.94 ± 0.38^c3^
JAFL	29.60 ± 7.42^f1^	40.08 ± 0.84^a2^	57.25 ± 0.25^b3^
Proso millet			
ANUL	38.74 ± 0.75^d1^	53.95 ± 0.29^d2^	25.25 ± 0.29^d3^
HAML	68.34 ± 2.67^e1^	27.79 ± 1.29^e2^	47.49 ± 4.42^e3^

Same letters in each column and same numbers in each raw are not significantly different (*p *>* *.05).

### 4.7 β ‐Carotene‐linoleate model system

In β **‐**carotene‐linoleate model system, the presence of phenolic compounds will hinder the extent of β carotene bleaching by neutralizing the linoleate free radicals and other free radicals formed within the system. Therefore, depending on the degree of antioxidant compounds present in the system retain the color of β carotene.

Table 5 presents the percentage of β carotene oxidation inhibition of soluble phenolic extracts of millets. The percentage of β carotene oxidation inhibition of soluble extracts of whole grains, dehulled grains, and hulls of finger millets ranged from 6.7 to 74.7, 11.5 to 63.1, and 31.8 to 91.7%, respectively. The percentage of β carotene oxidation inhibition of bound phenolic extracts of representative millet samples were presented in Table 7. This study results demonstrated that the millets inhibited the oxidation of β carotene to different degrees depending on the millet type, part of the seed, variety, and cultivated location. Chandrasekara and Shahidi (2010) reported that finger millet varieties (*Ravi* and local) and little millet had the highest antioxidant activity coefficient (AAC) in β carotene linoleate model system followed by pearl, kodo, foxtail, and proso millets. According to their results, bound phenolic extracts of kodo millet showed highest AAC followed by foxtail, little, finger (*Ravi*), proso, pearl, and finger millet local variety.

The soluble extracts of millet hulls and whole grains had highest percentage of β carotene oxidation inhibition compared to their counterparts of dehulled grains. The study conducted by Varsha et al. (2009) also showed a significant higher values (86%) for the seed coat extract in comparison to those of whole flour extract (27%) by the antioxidant activity determined by the β ‐carotene linoleate model system.

### 4.8 Ferrous ion chelating activity

Metal ions such as ferrous and cupric ions are the most effective prooxidant in biological systems. They are important catalysts for the generation of first few free radicals to initiate the radical‐mediated lipid peroxidation. Chelating agents are beneficial to inhibit the radical generation by stabilizing transition metal ions and subsequently reducing free radical damage. Phenolic compounds possess hydroxyl and carboxyl groups, having the ability to bind metal ions. Therefore, antioxidant activities of some phenolic compounds are due to their high tendency to chelate metal ions.

In this study, the ferrous ion chelating ability was measured by the formation of purple color complex of ferrous ions with ferrozine and the intensity of the purple color of the complex decreases in the presence of chelating agents. The soluble and bound phenolic extracts of millets showed different degrees of FICA. The FICA of soluble extracts of millets was in the order of finger > foxtail > proso millets (Table 6). The results of a previous study by Chandrasekara and Shahidi (2010) also showed that the phenolic compounds present in millets are good source of metal chelating agents to inhibit the radical‐mediated chain reactions. Their results showed that the FICA of soluble phenolic extracts of millets ranged from 0.37 to 7.99 μmol of EDTA equiv/g of defatted meal. They had obtained the highest metal chelating effect for the finger millets among the studied milllet samples (finger, proso, kodo, and foxtail millets).

In this study, the highest FICA was obtained for the dehulled grains compared to the whole grains and counterpart hulls of millets. The FICA demonstrated a positive relationship with PC (r = .472; *p* < .001) and DRSA (r = .572; *p* < .001) in dehulled grains of millets. Chandrasekara and Shahidi (2010) also presented that the FICA of soluble extracts of millets had a significant positive correlation with PC (r = .551; *p* < .01) and did not have significant correlation with TPC and TFC, which could be due to the formation of stable complexes by proanthocyanidins with metal ions as ferrous ion chelator. The highest FICA with more condensed tannin was found among the finger millet dehulled grains cultivated in dry zone (Agro‐ecological zones of DL1, 2, and 5) compared to those in intermediate zone in Sri Lanka.

### 4.9 HPLC analysis

Ferulic acid is a major hydroxycinnamic acid present in soluble and bound phenolic extracts of millets grains (Chandrasekara & Shahidi, 2011). Ferulic acid content of soluble phenolic extracts of whole grains, dehulled grains, and hulls of millet samples ranged from 33.3 to 366.9, 50.6 to 232.2, 299.5 to 3408.7 μg/g of dm, respectively (Table 8). Ferulic acid is a low‐molecular weight phenolic acid concentrated in the outer layers of cereal grains (Mueller‐Harvey, Harley, Harris, & Curzon, 1986). According to Rybka, Sitarski, and Raczynska‐Bojanowska (1993), rye grain flour and bran had 334 and 1684 μg of ferulic acid per g, respectively (Rybka et al., 1993). In finger millet, whole grain flour and hull consisted of 20 and 18 μg of ferulic acid per g, respectively (Varsha et al., 2009). The results of our study further confirmed the high ferulic acid content in millet hulls compared to their dehulled counterparts. The results of present study showed that 50%–98% contribution from bound fraction to the total ferulic acid content of whole grains of studied millet samples. The bound phenolic fraction contributed more ferulic acid to millet grains compared to their soluble counterparts. Previous studies also showed that higher contribution of ferulic acid from bound phenolic fractions compared to the soluble counterpart of millet grains (Chandrasekara & Shahidi, 2010, 2011; Mpofu et al., 2006; Zhang et al., 2014). Adom, Sorrells, and Liu (2003) showed that free, soluble conjugated, and bound ferulic acid contents of 11 studied wheat varieties were significantly different and contribution of bound ferulic acid was high as 97% to total ferulic acid content in all varieties.

**Table 8:** Ferulic acid content (μg / g of dry matter) of soluble and bound phenolic extracts of millets

** **	Whole grains		Dehulled grains		Hull	
	Soluble phenolics	Boundphenolics	Soluble phenolics	Boundphenolics	Solublephenolics	Boundphenolics
Finger millet						
MIRavi	107.88 ± 1.50^a1^	250.68 ± 0.45^a2^	82.75 ± 2.45^a1^	340.17 ± 1.25^a2^	3408.72 ± 2.50^a1^	3796.17 ± 0.52^a2^
MIRav	126.35 ± 1.42^b1^	760.85 ± 0.34^b2^	85.06 ± 2.00^a1^	284.15 ± 1.00^b2^	787.47 ± 2.54^b1^	1730.61 ± 1.40^b2^
MIOsh	171.69 ± 1.00^c1^	592.31 ± 0.50^c2^	101.78 ± 1.65^b1^	472.45 ± 0.56^c2^	1053.08 ± 3.5^c1^	2265.83 ± 3.60^c2^
Foxtail millet						
HAML	158.11 ± 0.56^d1^	674.76 ± 1.25^d2^	216.48±1.50^c1^	689.91 ± 0.35^d2^	575.29 ± 2.5^d1^	609.79 ± 0.57^d1^
MOL	153.29 ± 1.34^d1^	1522.55 ± 1.00^e2^	232.22 ± 1.45^c1^	269.55 ± 0.46^b2^	473.79 ± 2.53^e1^	1327.91 ± 0.45^e2^
JAFL	366.89 ± 1.45^e1^	403.27 ± 2.50^f1^	181.76 ± 1.56^d1^	1082.79±0.25^e2^	299.47 ± 4.5^f1^	433.79 ± 1.50^f2^
Proso millet						
ANUL	33.33 ± 0.56^f1^	290.30 ± 0.50^g2^	50.62 ± 0.56^e1^	349.64 ± 0.56^a2^	489.10 ± 0.56^e1^	1636.19 ± 1.55^g2^
HAML	34.76 ± 0.60^f1^	1942.38 ± 1.50^ h2^	54.97 ± 0.50^e1^	721.92 ± 0.50^f2^	730.26 ± 0.55^g1^	1335.04 ± 2.05^e2^

Same letters in each column and same numbers in each raw are not significantly different for each category (whole grains, dehulled grains, and hulls) (*p* > .05).

It is notewothy that a significant difference in ferulic acid content was observed in this study among finger millet varieties cultivated in the same location (Table 8). In a previous study, Rybka et al. (1993) showed that the contents of ferulic acid of three cultivars of rye grown under same environmental conditions were in the range of 1006 to 1138 μg per g of whole grain flour. Further, it has demonstrated a significant variation among the different barley cultivars (Zupfer, Churchill, Rasmusson, & Fulcher, 1998). Proso and foxtail millets cultivated at different locations also showed varying quantities of ferulic acid. In agreement, ferulic acid content was varied significantly in wheat cultivars grown in different environments (Abdel‐Aal et al., 2001).

The overall results of this study indicated that the phenolic contents and the antioxidant activities of millets were significantly affected by the variety and cultivated locations. The antioxidant activities with different mechanisms explain that millet grain phenolics can act in a number of ways against oxidative stress. Highest phenolic content and antioxidant activities were found in the studied millet samples obtained from dry zone in Sri Lanka. Since this is the first study conducted about the effect of growing conditions on phenolic contents and antioxidant activities of millets in Sri Lanka, the results may be important to optimize the growing conditions of selected variety to produce millets rich in natural antioxidants to combat against the burden of non‐communicable diseases arising in the country.

## Funding Information

This research was supported by the National Research Council of Sri Lanka (NRC 12‐096) through a research grant to AC.

## Conflict of Interest

None declared.
